# An Analysis of the Risk Factors for Postural Defects among Early School-Aged Children

**DOI:** 10.3390/jcm12144621

**Published:** 2023-07-11

**Authors:** Anna Baranowska, Matylda Sierakowska, Anna Owczarczuk, Beata Janina Olejnik, Agnieszka Lankau, Paweł Baranowski

**Affiliations:** 1 Department of Integrated Medical Care, Faculty of Health Sciences, Medical University of Bialystok, 15-096 Bialystok, Poland; matylda.sierakowska@umb.edu.pl (M.S.); agnieszka.lankau@umb.edu.pl (A.L.); 2 Rehabilitation Center Armedis, 15-032 Bialystok, Poland; annaowczarczuk@o2.pl; 3 Department of Developmental Age Medicine and Pediatric Nursing, Faculty of Health Sciences, Medical University of Bialystok, 15-295 Bialystok, Poland; beata.olejnik@umb.edu.pl; 4 Highschool No.19 of Sports Master, 15-720 Bialystok, Poland; pawelb.huzar@gmail.com

**Keywords:** postural defects, early school-aged children, lifestyle, physical activity, BMI

## Abstract

A considerable number of problems begin in childhood due to lifestyle changes, which include a transition from a previous period of extensive movement to prolonged hours of staying in a sitting position at school. The aim of this study was to examine the occurrence of back and side view postural defects in the study group of school-aged children and identify risk factors associated with the formation of postural abnormalities in the study group. Methods: This study was conducted on a group of 141 children aged 7–10, attending the first to third grades at a primary school in Białystok (northeastern Poland). This study involved measuring the children’s height and weight, assessing the children’s body posture based on the FITS method (Functional Individual Scoliosis Therapy) by Białek and M’hango (the authors of this study), and administering a diagnostic survey addressed to parents and guardians of the children (*n* = 104) using a self-designed questionnaire. Results: Almost all defects were more prevalent in boys, especially in the case of stature triangles (*p* = 0.0489) and knee alignment in the sagittal plane (*p* = 0.038). The age of the subjects differentiated the incidence of defects in the scapulae (*p* = 0.0037) and shoulder (*p* = 0.0129) alignment, correlating negatively with age. The risk of postural defects for knees (*p* = 0.0391) and abdominal arching (*p* = 0.0240) was significant with a higher BMI. The following lifestyle-related factors were significant: the seat for doing homework (stature triangles *p* = 0.0253), time spent in front of a computer (positioning of the scapulae in relation to each other *p* = 0.0233; vertical view of the intergluteal cleft *p* = 0.0324), and snacking between meals (feet *p* = 0.0003; shoulder positioning *p* = 0.0013; stature triangles *p* = 0.0186; positioning of the scapulae in relation to each other *p* = 0.0404). Conclusions: The body posture of the examined children was closed with the head pushed forward and drooped, rounded shoulders, hyperlordosis, and pelvic anteversion. Most exhibited various types of abnormalities related to the feet. The recognized risk factors for posture defects are overweight/obesity, the male gender, children who are older, lack of an adjustable work chair, 2 h a day or more spent using the computer, and snacking between meals.

## 1. Introduction

In recent years, abnormalities related to body posture, often referred to as postural defects, have been diagnosed more and more often in children. Numerous authors writing on the subject have tried to define what body posture is. It is believed that it is a way of holding the body in a standing position, which is characterized by optimal postural stability with minimal effort on the part of the muscular system. Posture is also a consequence of the arrangement of individual body segments in constant balance and is an indication of the physical and mental state [1]. It is characterized by variability and individualization of specific sections and, despite the similarities in the arrangement of individual body parts in relation to each other, it is identical in different individuals of the same sex and age [2,3].

It is impossible to clearly assess the frequency of posture defects. The discrepancies in the statements are most often related to a large number of definitions of individual abnormalities, the individual course of posturogenesis for each person, the criteria for assessing body posture, and many research methods [4]. In the literature, the estimated incidence of postural defects depends on the assessment method and the adopted criteria for correct body posture. Studies on the health of children and adolescents in Poland have shown that the incidence of posture defects ranges from 10 to 80% [5].

There are numerous causes of postural defects. Apart from cases of congenital defects or specific diseases, postural defects usually develop without any identifiable causes. Factors that cause the adoption of an incorrect body position play an important role. They perpetuate incorrect postural habits over time and contribute to the development of postural defects [3].

Both modifiable and non-modifiable factors influence the formation of body posture. The modifiable factors include body weight, the quality of spending free time, the use of electronic devices, the weight of the school bag, the way it is packed and worn, patterns from the environment, and the incorrect performance of exercises, as well as the psychophysical state of students [2].

Two school-age periods are particularly precarious in terms of the formation of quality of posture. They occur during the so-called growth spurts at 6–7 years of age and then at 12–16 years of age [3]. It should be noted that the need for physical activities for children in primary school is much greater than what is provided in the school curriculum. This mostly results from an insufficient number of hours of physical education and the lack of free time due to overburdening with learning, which results from research by Mańczak and Raciborski conducted in central Poland on a group of first-grade children [6]. The World Health Organization has recognized lack of exercise as the fourth leading risk factor for mortality among preventable diseases [7].

Postural defects are largely related to lifestyle changes. The once active lifestyle has become more sedentary, which seems to result from the more frequent use of ubiquitous electronic devices, such as computers or mobile phones. Students utilize these devices to perform both school duties (especially in the last years of the COVID-19 pandemic) and maintain social contacts, relax by playing games, and watch movies, and consequently, they often stay in their own rooms. According to the results of an international study on health behaviors in children and adolescents (Health Behavior in School-Aged Children (HBSC)), 44.4% of school children spend 4 h in front of a screen on school-free days [8].

Among the risk factors for postural defects, attention is also drawn to the overloaded school backpacks of most children and adolescents. Polish students drawing attention to this problem resulted in the Chief Sanitary Inspectorate issuing recommendations on the maximum load being 10–15% of the student’s body weight. However, opinions on the acceptable backpack weight and other elements that are taken into account are divided. Some take only the student’s body weight into account, and others advise the acceptable weight of the school bag to be dependent on gender or body mass index (BMI) [9,10,11]. In the United States of America, the recommended weight for a backpack is between 10 and 20% of a child’s body weight. In Europe, a weight above 10% is usually not acceptable. The lack of common international guidelines on the acceptable weight of schoolbags means that the problem has not been solved [11,12].

Another important factor associated with the formation of postural defects is obesity, which is one of the most serious health problems faced by children today [13]. Unfavorable relationships between excessive body weight and lack of physical activity and body posture disorders have been indicated [14]. In The State of the World’s Children 2019: Children, food, and nutrition, UNICEF reports that 40 million children are overweight or obese. Between 2000 and 2016, the number of overweight children aged 5 to 19 doubled (from 1 in 10 to almost 1 in 5). Currently, ten times as many girls and twelve times as many boys in this age group suffer from obesity in comparison to 1975 [15]. In Poland, according to the HBSC Report from 2018, every fifth child has excessive body weight. Overweight children are less physically active than their peers with normal weight. They participate in physical education classes and other extracurricular forms of physical activity less often [14]. Overloading the musculoskeletal system with excessive weight and motor passivity results in improper development of body posture patterns in the period of posturogenesis. In particular, these changes concern the sagittal plane and knee and foot defects. In adult life, these consequences are manifested in but not limited to the occurrence of pain in the area of the vertebral column [16].

Preventing the development of postural abnormalities by eliminating the factors conducive to their development and ensuring that children maintain proper body posture is within the scope of prophylaxis. Taking the above-mentioned factors into account, attention should be paid to activities preventing abnormalities and ensuring children’s participation in screening tests from the earliest age [8,17]. In order to detect signs indicating abnormalities in body posture, it is necessary to cooperate with a team of people who are closest to a child of pre-school and school age, i.e., an aware parent, school nurse, physical education teacher, and physiotherapist [3]. A delayed response may lead to persistent contractures and, consequently, deformations of the osteoarticular system, resulting in serious health disorders [2].

The goal of this study was to identify postural defects among early school-aged children and determine their type, as well as identify risk factors related to the occurrence of abnormalities in body posture. For this purpose, we aimed at identifying the relationship between the occurrence of postural defects and gender, age, BMI (WHO—z-score), the weight of school bag/backpack, the place for doing homework, and the broadly understood lifestyle of students (way of spending free time, scope of physical activity, and nutrition).

## 2. Material and Methods

### 2.1. Participants

The study involved 141 children who were 8, 9, and 10 years of age. The study group consisted of students at a primary school in Białystok (northeastern Poland) and their parents/guardians (141 questionnaires were distributed, and 104 were gathered and included in the analysis).

### 2.2. The School Environment of the Participating Children

The participating students attended the 1st, 2nd, or 3rd grade. The number of lesson hours according to the core curriculum is about 24 lessons per week, including 3 h of physical education. Classes were not conducted by a physical education teacher but by a form teacher and took place in the school corridor. The first and second grades spent one hour a week at the swimming pool. Each class had its own classroom where educational activities were usually held. The school desks at which the students sat were one size. The children were allowed to leave some textbooks in the classroom, so as not to carry them home and back. During our research, children had a hybrid learning system so during in-class education, half of the children were spending approximately 1 h in front of the computer; however, during remote classes, they were spending 4–5 h a day.

### 2.3. Study Design and Data Collection

The research was carried out from March to June 2022. The project involved examining the children’s body posture, as well as conducting a survey of their parents to determine the children’s lifestyle. Data analysis was used to ascertain the determinants of postural defects. The parents gave their written consent to the examination of the children and the questionnaire. Participation in the study was voluntary. The research was anonymous, and each participant could withdraw from the study at any time. Participation in the study was tantamount to consenting to it.

### 2.4. Measurements

Inclusion and exclusion criteria were used to create a homogeneous group. The inclusion criteria were age 8–10, presence at school on the day of the examination, and written parental consent for the examination. The exclusion criteria were musculoskeletal system diseases affecting body posture, genetic defects affecting the musculoskeletal system, systemic diseases, joint and muscle diseases that are a contraindication to the examination, significant vision and hearing defects, skin diseases, post-traumatic conditions of the musculoskeletal system, and the malaise of the child on the day of the examination.

The study was conducted by a team consisting of a physiotherapist and a nurse in the office of the school nurse. The examination was performed in the morning, during school hours.

The examination of the children consisted of:

1. Measuring the children’s height (standard measurement in centimeters) and weight (electronic scale). BMI was calculated, and BMI values were classified in accordance with the WHO standards from 2007 [18]. Z-score values and BMI classification into four categories were determined: underweight (z-score below −2), normal weight (z-score between −2 and 1), overweight (z-score above 1), and obese (z-score above 2).
2. Assessing the body posture of children, a questionnaire based on the FITS method (Functional Individual Therapy of Scoliosis) by Białek and M’hango [19] was used. A ruler, a plumb line, and a scoliometer were used to examine body posture. The examination involved inspecting the following:
  - The frontal plane: head and neck, shoulder alignment, positioning of the scapulae relative to each other, positioning of the scapulae relative to the vertebral column (distance), the vertebral column, stature triangles, and vertical view of the intergluteal cleft, pelvis, knees, and feet.
  - The sagittal plane: head, shoulders, abdominal arching, the shape of the spine in the sagittal plane, pelvis, and knees.

The second part of the research process consisted of a survey of parents/guardians. The study used the diagnostic survey method. The research tool was a self-constructed questionnaire consisting of 4 metric questions and 20 detailed questions concerning the topic of the study. In the questionnaire, parents were asked, among other things, their living conditions, knowledge about the prevention of postural defects, and the factors that may pose a risk to the correct posture of children. These included the way of reaching school, the setting in which homework is done, time spent studying at the computer, physical activity, free time, and eating habits of their children.

## 3. Ethical Consideration

The research was carried out in accordance with the Declaration of Helsinki and Good Clinical Practice in research. The Bioethics Committee of the Medical University in Bialystok, Poland, granted the ethical approval for the study: SUB/3/NN/22/002/3310. Participation was voluntary and participants were informed about the project.

## 4. Statistical Analysis

The descriptive part contains information on the frequency of postural abnormalities and the general characteristics of the surveyed population. The significance of differences in the occurrence of postural defects in relation to selected factors (e.g., child’s sex, time spent at the computer, ergonomics of doing homework) was verified using the chi-square test, and differences between the total number of postural abnormalities were assessed using the Mann–Whitney U test (for two groups) or the Kruskal–Wallis test for a larger number of compared groups.

If the independent variable was numerical, such as a BMI z-score or the weight of the backpack worn to school, its correlation with the occurrence of particular postural abnormalities was measured using odds ratios determined using single-factor logistic regression models. The relationship between these factors and the number of postural abnormalities was tested using Spearman’s rank correlation coefficient.

The results of the statistical tests were interpreted according to common guidelines, assuming a result was statistically significant at *p* < 0.05. When the *p*-value slightly exceeded 0.05, the results were included in the discussion, but it was emphasized that the reliability of these conclusions should be the subject of further research.

A statistically significant correlation was assumed at *p* < 0.05 (*); *p* < 0.01 is a highly significant correlation (**); and *p* < 0.001 is a very highly statistically significant correlation (***).

## 5. Results

### 5.1. General Characteristics of the Study Group

The study involved 141 children aged 8–10, among whom 58 (41%) were girls and 83 (59%) were boys. The children attended primary school; 43 of them (31%) attended 1st grade, 58 (41%) 2nd grade, and 40 (28%) attended 3rd grade. Knowing the children’s height and weight, BMI was calculated and z-score values were determined, including the BMI classification into four categories: underweight, normal weight, overweight, and obese.

Almost every third child had a higher-than-normal body weight (30.5%) because every tenth student was classified as obese (9.9%) and every fifth student was classified as overweight (20.6%). On the other hand, the weight of 67.4% of the respondents was within the norm.

The average BMI z-score was slightly higher than 0, which means that the children in the study group had a body weight slightly higher than the norm. This is particularly evident among 8-year-old children (z-score for boys was 0.45 and 0.36 for girls) (data in Table 1).

### 5.2. Postural Defects in the Study Group

After examining postural defects among the students, the most common irregularities were differentiated. In the coronal plane, there were foot abnormalities (70%), especially collapsed longitudinal arches of the foot (48.9%), valgus (22%), and flat feet (19.1%). Subsequently, abnormalities in the scapulae and shoulders were identified. Scapular asymmetry occurred in 86 students (61%), with the left scapula typically superior (35.5%) and the right scapula more lateral to the vertebral column (27%). In 68 patients (48.2%), there was a lack of symmetry in the position of the shoulders, with the left one frequently situated superiorly (27.7%). Almost half of the students (46.1%) had asymmetrical stature triangles, and in every third student (34%), the right triangle had a larger surface area. Pelvic obliquity was found in 39.7% of the children. Scoliotic posture affected 40 (28.4%) children. 

Examination of the sagittal position when viewing the silhouette from the side showed that the most frequently observed abnormality was rounded shoulders (72.3%) and forward head posture in 71 (50.4%) subjects. Similarly, almost half (42.6%) showed signs of anterior pelvic tilt. Only every third child (36.9%) had the correct shape of the vertebral column. The most common diagnoses were hyperlordosis (24.1%) and flat back (18.4%), and every 10th person had a round concave back (10.6%) and a round back (9.9%). 

It is noteworthy that only one child among the subjects did not have any body postural defects. The majority (75%) had at least several abnormalities.

### 5.3. Factors Differentiating the Incidence of Postural Defects

An attempt was made to differentiate the occurrence of postural defects depending on the sex and age of children, as well as the school backpack weight. The correlation between BMI and the occurrence of posture defects was also considered. Both specific postural defects and their total number were analyzed.

#### 5.3.1. Sex and the Incidence of Postural Defects

The postural defects presented in Table 2 were ranked according to the frequency of occurrence in the entire population. In general, there was a higher incidence of posture defects in boys. Significant differences between girls and boys concerned abnormalities of the waist triangles (*p* = 0.0489) and knees (*p* = 0.0388) in the sagittal plane. In both cases, defects were more common among boys.

#### 5.3.2. Age and the Incidence of Postural Defects

As shown in Table 3, age is a factor that significantly differentiates the incidence of postural defects in the scapular (*p* = 0.0037) and shoulder (*p* = 0.0129) region, and there is a noticeable negative correlation with age.

#### 5.3.3. BMI and the Incidence of Postural Defects

The impact of the BMI z-score on the probability of a given postural defect was assessed based on the BMI z-score values. For this purpose, logistic regression models were utilized. The value of the odds ratio (OR) demonstrates how many times the chance of a given postural defect in a child increases (when OR > 1) or decreases (when OR < 1) when the BMI z-score increases by 1. The data gathered from 16 logistic regression models shows that a higher BMI increases the risk of postural defects in the knees viewed from the frontal plane (*p* = 0.0391) and abdominal arching (*p* = 0.0026) (data in Table 4).

The weight of the school bag and the incidence of postural defects.

In order to find risk factors for postural defects, children’s school backpacks were weighed on the day of the examination. The Chief Sanitary Inspectorate in Poland recommends that the weight of the schoolbag should not exceed 10–15% of the child’s body weight. The following classification was made on the basis of this recommendation. We found that 60.2% of the surveyed students had a schoolbag weighing more than 10% of their body weight.

It was noted that the male first graders had the heaviest schoolbags. There is a certain tendency for the weight of the schoolbag to decrease in the later grades.

While the average weight of the schoolbags of 8-year-old girls is 3.7 kg and 4.0 kg for boys, it is, respectively, 3.6 and 3.6 kg for children one year older, and 3.2 and 3.1 kg for 10-year-old children (data in Table 5).

Using logistic regression models, the potential impact of schoolbag weight on the incidence of individual postural defects was investigated. The conducted analyses demonstrate that the greater weight of the school bag is not related to the risk of postural defects (*p* > 0.05).

### 5.4. Factors Differentiating the Incidence of Postural Defects Related to the Student’s Lifestyle

In order to find determinants of postural defects, parents were asked about the broadly understood lifestyle of their children. Women (mothers/guardians) were the most likely to speak about their children (81.7%).

The respondents were asked about the living conditions to assess the parents’ ability to provide conditions for the proper development of children. These conditions were reported to be good (42.3%) or very good (48.1%). The majority of respondents (66.3%) rated their knowledge of the prevention of postural defects as average. Every sixth person (16.3%) admitted to a complete lack of knowledge of the symptoms of incorrect posture.

We analyzed the factors that may pose a risk to the correct posture of the children. These included the way of reaching school, the setting in which homework is done, time spent studying at the computer, physical activity, free time, and eating habits of the children.

According to information provided by the parents, the majority of the children (>58%) reach school on foot, and the remaining children are usually transported by car. The conducted analyses showed that the way of reaching school was not correlated in a statistically significant way with the occurrence of postural abnormalities, both for individual abnormalities and their total number (*p* > 0.05).

More than half (56.7%) of the surveyed students had their own room, a desk where they did their homework (80%), and an adjustable chair (70.2%). Despite these seemingly favorable conditions, the influence of the type of seat used for doing homework on the incidence of postural defects was studied. It was discovered that children who did not have an adjustable chair had a greater chance (34% vs. 58%) of developing abnormalities in the area of stature triangles (*p* = 0.0253) (data in Table 6).

Another factor in children’s learning was the amount of time spent at the computer.

More than half (52.9%) of the children spend less than one hour a day studying in front of the computer, and every third child (38.5%) spends up to three hours a day. The analyses demonstrated that children who spend more time at the computer studying had significantly more defects in scapular alignment (*p* = 0.0233) and vertical view of the intergluteal cleft (*p* = 0.0324) (Table 7).

Overall, children who spend more time at the computer had, on average, more defects in the frontal plane (4.3 vs. 3.4) (*p* = 0.0552) and thus a greater number of total defects (7.0 vs. 6.0); however, it was not statistically significant (*p* = 0.0769).

As far as physical activity is concerned, almost all children (96.2%) participated in physical education classes and additional physical activities (76.9%). Almost half (43.3%) of the subjects did so 2–3 times a week. Children also spend a significant amount of free time outside (31.7%, from 3 to 5 h). However, almost every third child also used a mobile phone (31.7%), watched TV (30.8%), or used a computer (29.8%) in their free time.

Nutrition is another risk factor for postural defects in children. The information provided by the parents shows that most children (81.7%) ate 4–5 meals a day and fruit 1–2 times a day. At the same time, the vast majority (76.9%) snacked between meals. Every fifth child (17.3%) ate fast food and half of the subjects ate at least small amounts of sweets every day.

Correlations between snacking between meals and the occurrence of postural defects, and then between snacking and BMI (z-score BMI), were studied. This revealed that children who snacked between meals had significantly more abnormalities in shoulder alignment (*p* = 0.0013), scapular alignment (*p* = 0.0404), and stature triangles (*p* = 0.0186). Children who snacked less often or not at all had significantly more foot defects (*p* = 0.0003) (data in Table 8).

When correlating the BMI z-score to snacking between meals, no statistically significant differences were found (*p* > 0.05).

## 6. Discussion

Postural defects in children have been the subject of many studies for several years. It is estimated that they affect from 30 to 80% of the examined children. Due to the nature of the problem, primary healthcare finds prevention, early diagnosis, and treatment of postural defects, as well as prevention and treatment of overweight and obesity in children in early school age, to be of great importance [20].

Children aged 8–10, i.e., in a specific risk group, in whom the probability of postural defects increases with the beginning of their school education, participated in our study. This is mainly due to the transition to a more sedentary lifestyle, which significantly affects the skeletal system. Therefore, the aim of this study was to determine the occurrence and the type of abnormalities in the body posture of children and identify the risk factors. The analyses showed that only one child among the examined students did not have any defects. The majority (75%) were diagnosed with at least some abnormalities. Upon examination in the frontal plane, the most common abnormalities affected the feet, including the most frequently collapsed longitudinal arch of the foot, valgus, and flat feet. Almost half had scapular and shoulder asymmetry, asymmetric stature triangles, and a tilt in the pelvis.

The literature on the research conducted among Bulgarian children also indicates that postural defects were present in 62% of early school-age children [21]. On the other hand, in Slovak studies, abnormalities in body posture were found in half of the examined children. Deviations in shoulders and scapulae exceeded 80%, and flat feet affected 65% of the children. In almost half of the cases, pelvis anteversion and head protrusion were also observed [22]. Slightly different results were obtained by other authors, where 51.5% of girls and 64.1% of boys were not diagnosed with posture defects [23]. Similarly, Kochman reports that 76% of children aged 11–14 had the correct body posture. Conversely, flat feet were found in 11% of the subjects [24]. A similar incidence of flat feet was observed in Chilean studies [25]. On the other hand, flat feet occurred in Bulgarian children in every fourth child, which is consistent with our research [21].

In current studies, the most common sagittal plane abnormality was rounding of the shoulders (>72%). Similar results were obtained by Kratenová, who observed this problem in every second student [26]. In addition, in our study, half of the students showed signs of head protrusion in a relaxed position and an abnormal shape of the vertebral column and anterior pelvic tilt. Scoliotic posture affected almost 1/3 of the respondents, similar to the study by Bueno, R. C. et al. among children in southern Brazil [27].

Nearly all abnormalities diagnosed in our research were observed more often in boys, although sex significantly differentiated the occurrence of postural defects only in stature triangles and knees in boys. In other Polish studies, where incorrect body posture was found in about 42% of children, defects were also more common in boys. The body posture of boys was more often characterized by hyperkyphosis and lumbar hyperlordosis in girls [28].

Excess weight, which can be observed in younger children, is an additional burden for body posture. Childhood obesity is a significant problem worldwide. Studies show that over forty million children under the age of 5 are overweight or obese [29]. As Lanigan writes in his article, in England, the last national measurement survey of children showed that almost every fourth child surveyed at the beginning of their school education was already obese [30]. This is a serious problem because such early obesity is difficult to reverse in later childhood and adulthood [31,32]. 

In our research, based on the BMI (WHO z-score) classification, the examined group of children was mostly within the normal weight range (>67%), but every tenth student was classified as obese, and every fifth student was overweight. The applied logistic regression models indicated that the higher the BMI, the greater the risk of postural defects in the area of knees and abdominal arching.

The importance of the problem of obesity is emphasized in the reports by Brzeziński [33], who indicates that postural defects most often affected obese children (>90%). A higher risk of postural defects of the lower limbs was correlated with weight gain. Gijon-Nogueron et al. also studied the relationship between obesity and foot positioning in children; however, they indicated that body weight, as well as sex and age, did not significantly affect foot positioning [34]. On the other hand, a Spanish study demonstrated that an increase in body weight was associated with a lowering of the medial longitudinal arch of the foot, which was lower in obese children [35]. Similar results were obtained by Brzęk, who compared a group of obese children aged 9–13 with a group of normal weight children. Significantly more postural abnormalities were found in obese children [14]. This was also confirmed by Maciałczyk-Paprocka et al., who showed that in a group of students aged 7–12, the probability of postural defects was much higher among obese students. The most common postural deviations in obese children and adolescents were valgus knees and flat feet [36]. A higher incidence of flat feet was also found in obese Chilean children in comparison to children with overweight and normal weight [25]. Purenovic reviewed international studies on postural defects, which, despite methodological differences, showed the negative impact of obesity on posture in children, even though it was not the most significant and basic risk factor [37].

There is a substantial body of research on the impact of the load of a school backpack on children. An analysis was performed by Australian authors [38]. They studied the impact of the type of backpack and the way it was carried or pulled [39], as well as the contents of school backpacks, and whether the weight of the backpack correlated to sex and age [11,40]. The influence of the load of a school backpack on body posture, foot load, and gait was also the subject of research [41]. Back pain levels were also measured. Another study, conducted by Spiteri et al. in all schools in Malta, showed that among pupils aged 8–13 (4005 participants), more than 70% had a backpack that exceeded the recommended backpack weight to body weight ratio of 10%. Additionally, a third of the children complained of back pain. The presence of back pain was statistically related to gender, body mass index (BMI), and backpack weight-to-body weight ratio [42].

The reports of Polish researchers are alarming. They indicate that more than 1/3 of primary school students have schoolbags exceeding 15% of the children’s body weight. The heaviest backpacks, in relation to body weight, were worn by more than half of the surveyed first-grade students [43]. Similarly, in our research, the youngest children had the heaviest backpacks. However, no strong, statistically significant correlations were found between the weight of a school bag and the incidence of postural defects.

One of the risk factors analyzed in our study was the use of an adjustable chair by a child when doing homework. It turned out that children who did not use an adjustable chair had a higher risk of postural defects related to stature triangles. Mrozkowiak and Żukowska also studied the impact of a “good chair” in the process of correcting static body postural misalignments. The authors concluded that an ergonomic seat plays an important and complementary role in the prevention of posture defects in children aged 7–10 [44].

Computer usage is one more factor contributing to the risk of postural defects. It is not difficult to notice that the availability of electronic devices has increased significantly in recent years. Their use among children is ubiquitous. In addition, positions other than sitting while studying may contribute to abnormalities in body posture [17,45,46]. It seems that, nowadays, it is impossible to function without electronics; however, one needs to be aware of their proper use. Our research shows that more than half of the children spend up to an hour a day in front of a computer while studying, and every third child spends up to three hours a day. Defects related to the positioning of the scapulae in relation to each other and the vertical view of the intergluteal cleft were significantly more common in children who spend more time using a computer. Similarly, other studies show that every fourth child spends up to sixty minutes, almost every second child spends more than one hour, and every third child spends more than two hours in front of a computer. Boys used the computer more often [13].

Participation in physical activities is another important aspect of the proper psycho-physical development of a child. The Health Behavior in School-aged Children (HBSC) report from 2018 shows that the level and intensity of physical activity undertaken by children in Poland has statistically decreased compared to 2014. Only 17.8% of the surveyed students met the required level of physical activity, which the WHO defines as at least 60 min of moderate- or high-intensity exercise per day [47,48].

Our research shows that the level of activity of children was rather high because almost all children participated in physical education classes, although it was available for only three lesson hours, i.e., 135 min a week. Almost 77% of the surveyed children participated in extracurricular physical activities and almost half did so 2–3 times a week. Similarly, in Kochman’s research, most students regularly participated in physical education classes. Every third child spends time in a physically active manner for up to two hours a day, and every fifth child did so for more than two hours a day [24]. A Czech study demonstrates that children spend 4 h a week participating in various sports activities. It was shown that 20% of those who did not participate in physical activities were more at risk of abnormal posture than children who regularly participated in sports activities [26]. In other studies, where the activity of school-age children was also assessed, it was shown that regular physical activity of children at a younger school age, at least three times a week, assists in the prevention of postural abnormalities in the torso [49].

In summary, the problem of postural abnormalities in children is widespread. It is not so much a hereditary issue as it is a result of the changing lifestyles of both children and their parents: the world of electronics, sedentary lifestyles, and little physical activity. This also includes a fast-food diet, a fast-paced lifestyle, stress, lack of time, and associated lifestyle diseases, such as obesity, which affects an increasing number of children at a younger age [25].

### 6.1. Conclusions

The body posture of the children that emerged in our research is a closed posture with a protruding head, protruding and rounded shoulders, hyperlordosis, and a forward tilt. In addition, most children have various types of foot abnormalities, which bodes poorly for the future, as dysfunction of the feet can lead to further asymmetry. Almost all of the examined children have postural defects of varying degrees, and most have several.

Only some of the identified postural defects are significantly dependent on the examined risk factors, such as age (older children), gender (more frequent in boys), BMI (overweight/obesity), study desk seating (lack of an adjustable chair), time spent at a computer (two hours or more), and nutrition (snacking between meals).

### 6.2. Practical Recommendations

Considering the negative changes in lifestyle over the past several years and an increase in mental health problems in children, it can be assumed that unaddressed postural defects will worsen. This may generate numerous health problems related not only to the musculoskeletal system in the coming years. It seems that further research is needed to determine the causes of such a large number of postural defects among children. A strong emphasis should be placed on screening tests, health promotion, and early prevention.

## 7. Limitations

This study certainly has limitations. Firstly, the students comprising the study group were from a selected macro-region, which does not allow for a generalization of the results to the entire Polish population. Secondly, the research on the group of parents/guardians was cross-sectional, based solely on self-assessment. Despite these limitations, the results of this study can serve as a starting point for further research on the impact of the school environment and student lifestyle on the risk of postural abnormalities in early school-age children. This also indicates the direction of the goals and tasks that the healthcare and education systems face in taking preventive or corrective actions, as well as educational measures for children and parents/guardians.

## Figures and Tables

**Table 1:** Z-score BMI.

Sex	Age											
	8 Years				9 Years				10 Years			
	$\bar{X}$	s	Min	Max	$\bar{X}$	s	Min	Max	$\bar{X}$	s	Min	Max
z-score BMI												
Female	0.36	1.08	−0.97	2.85	−0.03	1.39	−3.02	2.50	−0.08	1.43	−1.99	2.36
Male	0.45	1.23	−2.08	2.29	0.29	1.22	−1.71	2.62	0.27	1.41	−3.91	2.45

**Table 2:** The incidence of abnormalities in body posture depending on sex.

Incidence of Abnormalities	Sex				*p*-Value
	Female		Male		
	*n*	%	*n*	%	
Shoulders (B)	41	70.7%	62	74.7%	0.5975
Feet (A)	41	70.7%	59	71.1%	0.9595
Vertebral column shape (B)	34	58.6%	55	66.3%	0.3546
Positioning of the scapulae relative to each other (A)	34	58.6%	52	62.7%	0.6293
Head (B)	27	46.6%	44	53.0%	0.4503
Shoulder alignment (A)	26	44.8%	42	50.6%	0.4995
Positioning of the scapulae relative to the vertebral column (A)	26	44.8%	41	49.4%	0.5928
Stature triangles (A)	21	36.2%	44	53.0%	0.0489 *
Pelvis (B)	25	43.1%	35	42.2%	0.9120
Pelvis (A)	19	32.8%	39	47.0%	0.0911
Vertebral column (A)	15	25.9%	26	31.3%	0.4821
Abdominal arching (B)	13	22.4%	23	27.7%	0.4778
Vertical view of the intergluteal cleft (A)	13	22.4%	21	25.3%	0.6933
Knees (B)	5	8.6%	18	21.7%	0.0388 *
Head and neck (A)	5	8.6%	15	18.1%	0.1134
Knees (A)	6	10.3%	8	9.6%	0.8902

*p*—the probability values calculated using the chi-square independence test. *p* < 0.05 level was assumed as a statistically significant relationship (*). A—frontal plane. B—sagittal plane.

**Table 3:** The incidence of abnormalities in body posture depending on age.

Incidence of Abnormalities	Age						*p*-Value
	7 Years		8 Years		9 Years		
	*n*	%	*n*	%	*n*	%	
Shoulders (B)	34	79.1%	40	69.0%	29	72.5%	0.5249
Feet (A)	33	76.7%	40	69.0%	27	67.5%	0.5941
Vertebral column shape (B)	32	74.4%	33	56.9%	24	60.0%	0.1746
Positioning of the scapulae relative to each other (A)	19	44.2%	35	60.3%	32	80.0%	0.0037 **
Head (B)	20	46.5%	28	48.3%	23	57.5%	0.5568
Shoulder alignment (A)	19	44.2%	22	37.9%	27	67.5%	0.0129 *
Positioning of the scapulae relative to the vertebral column (A)	21	48.8%	24	41.4%	22	55.0%	0.4057
Stature triangles (A)	17	39.5%	26	44.8%	22	55.0%	0.3572
Pelvis (A)	18	41.9%	23	39.7%	17	42.5%	0.9548
Pelvis (B)	18	41.9%	23	39.7%	17	42.5%	0.9548
Vertebral column (A)	13	30.2%	15	25.9%	13	32.5%	0.7612
Abdominal arching (B)	11	25.6%	18	31.0%	7	17.5%	0.3197
Vertical view of the intergluteal cleft (A)	10	23.3%	14	24.1%	10	25.0%	0.9829
Knees (B)	5	11.6%	11	19.0%	7	17.5%	0.5970
Head and neck (A)	8	18.6%	4	6.9%	8	20.0%	0.1146
Knees (A)	5	11.6%	4	6.9%	5	12.5%	0.5973

*p*—the probability values calculated using the chi-square independence test. *p* < 0.05 level was assumed as a statistically significant relationship (*); *p* < 0.01 is a highly significant relationship (**) A—frontal plane. B—sagittal plane.

**Table 4:** The risk of postural defects and BMI values.

Postural Defects	z-Score BMI	
	OR (95% p.u.)	*p*-Value
Head and neck (A)	1.304 (0.889–1.913)	0.1745
Shoulder alignment (A)	1.040 (0.803–1.346)	0.7676
Positioning of the scapulae relative to each other (A)	0.954 (0.732–1.243)	0.7250
Positioning of the scapulae relative to the vertebral column (A)	0.817 (0.628–1.064)	0.1340
Vertebral column (A)	0.907 (0.683–1.206)	0.5030
Stature triangles (A)	0.726 (0.553–0.954)	0.0216 *
Vertical view of the intergluteal cleft (A)	0.914 (0.676–1.236)	0.5605
Pelvis (A)	0.811 (0.620–1.060)	0.1246
Knees (A)	1.648 (1.025–2.647)	0.0391 *
Feet (A)	1.192 (0.895–1.588)	0.2304
Head (B)	1.071 (0.827–1.387)	0.6052
Shoulders (B)	0.608 (0.440–0.840)	0.0026 **
Abdominal arching (B)	1.438 (1.049–1.970)	0.0240 *
Vertebral column shape (B)	0.847 (0.645–1.112)	0.2316
Pelvis (B)	1.041 (0.802–1.352)	0.7633
Knees (B)	0.691 (0.481–0.993)	0.0458 *

*p* < 0.05 level was assumed as a statistically significant relationship (*); *p* < 0.01 is a highly significant relationship (**). A—frontal plane. B—sagittal plane.

**Table 5:** School bag/backpack weight (kg) depending on age.

Sex	Age											
	8 Years				9 Years				10 Years			
	$\bar{x}$	*s*	Min	Max	$\bar{x}$	*s*	Min	Max	$\bar{x}$	*s*	Min	Max
School bag/backpack weight (kg)												
Female	3.66	0.82	1.75	4.90	3.59	0.79	1.80	5.50	3.18	0.88	1.70	4.60
Male	4.00	0.96	1.75	5.80	3.58	0.66	2.05	4.85	3.14	0.94	1.55	5.40

**Table 6:** The incidence of abnormalities depending on the type of chair used.

Incidence of Abnormalities	Type of Chair Used				*p*-Value
	Adjustable Chair (*n* = 70)		Other (*n* = 29)		
	*n*	%	*n*	%	
Head and neck (A)	9	12.9%	4	13.8%	0.9001
Shoulder alignment (A)	34	48.6%	13	44.8%	0.7342
Positioning of the scapulae relative to each other (A)	42	60.0%	18	62.1%	0.8480
Positioning of the scapulae relative to the vertebral column (A)	33	47.1%	14	48.3%	0.9182
Vertebral column (A)	18	25.7%	11	37.9%	0.2242
Stature triangles (A)	24	34.3%	17	58.6%	0.0253 *
Vertical view of the intergluteal cleft (A)	16	22.9%	8	27.6%	0.6173
Pelvis (A)	23	32.9%	15	51.7%	0.0790
Knees (A)	7	10.0%	2	6.9%	0.6250
Feet (A)	53	75.7%	20	69.0%	0.4874
Head (B)	37	52.9%	15	51.7%	0.9182
Shoulders (B)	50	71.4%	21	72.4%	0.9211
Abdominal arching (B)	14	20.0%	8	27.6%	0.4086
Shape of the vertebral column (B)	40	57.1%	19	65.5%	0.4397
Pelvis (B)	32	45.7%	14	48.3%	0.8161
Knees (B)	11	15.7%	5	17.2%	0.8510

*p* < 0.05 level was assumed as a statistically significant relationship (*). A—frontal plane. B—sagittal plane.

**Table 7:** The incidence of abnormalities in body posture depending on the time spent at a computer.

Incidence of Abnormalities	Time Spent at a Computer (Classroom Instruction)				*p*-Value
	Up to 1 h (*n* = 52)		2 h or More (*n* = 44)		
	*n*	%	*n*	%	
Head and neck (A)	5	9.6%	8	18.2%	0.2216
Shoulder alignment (A)	23	44.2%	22	50.0%	0.5725
Positioning of the scapulae relative to each other (A)	26	50.0%	32	72.7%	0.0233 *
Positioning of the scapulae relative to the vertebral column (A)	22	42.3%	22	50.0%	0.4510
Vertebral column (A)	11	21.2%	16	36.4%	0.0986
Stature triangles (A)	18	34.6%	21	47.7%	0.1925
Vertical view of the intergluteal cleft (A)	8	15.4%	15	34.1%	0.0324 *
Pelvis (A)	17	32.7%	19	43.2%	0.2902
Knees (A)	5	9.6%	4	9.1%	0.9300
Feet (A)	40	76.9%	32	72.7%	0.6362
Head (B)	26	50.0%	24	54.5%	0.6569
Shoulders (B)	39	75.0%	29	65.9%	0.3289
Abdominal arching (B)	13	25.0%	8	18.2%	0.4207
Shape of the vertebral column (B)	29	55.8%	28	63.6%	0.4342
Pelvis (B)	24	46.2%	22	50.0%	0.7070
Knees (B)	8	15.4%	7	15.9%	0.9438

*p* < 0.05 level was assumed as a statistically significant relationship (*). A—frontal plane. B—sagittal plane.

**Table 8:** The incidence of postural abnormalities and snacking between meals.

Incidence of Abnormalities	Snacking between Meals						*p*-Value
	Yes (*n* = 25)		Sometimes (*n* = 52)		No (*n* = 20)		
	*n*	%	*n*	%	*n*	%	
Head and neck (A)	4	16.0%	7	13.5%	1	5.0%	0.5058
Shoulder alignment (A)	19	76.0%	21	40.4%	5	25.0%	0.0013 **
Positioning of the scapulae relative to each other (A)	20	80.0%	29	55.8%	9	45.0%	0.0404 *
Positioning of the scapulae relative to the vertebral column (A)	15	60.0%	23	44.2%	7	35.0%	0.2228
Vertebral column (A)	11	44.0%	10	19.2%	6	30.0%	0.0737
Stature triangles (A)	16	64.0%	17	32.7%	6	30.0%	0.0186 *
Vertical view of the intergluteal cleft (A)	6	24.0%	13	25.0%	4	20.0%	0.9043
Pelvis (A)	11	44.0%	20	38.5%	5	25.0%	0.4054
Knees (A)	2	8.0%	3	5.8%	4	20.0%	0.1703
Feet (A)	11	44.0%	45	86.5%	16	80.0%	0.0003 ***
Head (B)	12	48.0%	26	50.0%	12	60.0%	0.6880
Shoulders (B)	19	76.0%	33	63.5%	17	85.0%	0.1611
Abdominal arching (B)	9	36.0%	7	13.5%	5	25.0%	0.0734
Shape of the vertebral column (B)	16	64.0%	27	51.9%	14	70.0%	0.3121
Pelvis (B)	11	44.0%	23	44.2%	11	55.0%	0.6869
Knees (B)	3	12.0%	9	17.3%	3	15.0%	0.8319

*p* < 0.05 level was assumed as a statistically significant relationship (*); *p* < 0.01 is a highly significant relationship (**); *p* < 0.001 is a very highly statistically significant relationship (***). A—frontal plane. B—sagittal plane.

## Data Availability

Data not publicly available.
